# Topical Diclofenac Reprograms Metabolism and Immune Cell Infiltration in Actinic Keratosis

**DOI:** 10.3389/fonc.2019.00605

**Published:** 2019-07-03

**Authors:** Katrin Singer, Katja Dettmer, Petra Unger, Gabriele Schönhammer, Kathrin Renner, Katrin Peter, Peter J. Siska, Mark Berneburg, Wolfgang Herr, Peter J. Oefner, Sigrid Karrer, Marina Kreutz, Elisabeth Datz

**Affiliations:** ^1^Department of Internal Medicine III, University Hospital Regensburg, Regensburg, Germany; ^2^Institute of Functional Genomics, University of Regensburg, Regensburg, Germany; ^3^Department of Dermatology, University Hospital Regensburg, Regensburg, Germany; ^4^Regensburg Center for Interventional Immunology, University of Regensburg, Regensburg, Germany

**Keywords:** actinic keratosis, diclofenac, CD8, CD1a, metabolism, lactate

## Abstract

**Background:** Melanoma and squamous cell carcinoma of the skin are characterized by an altered glucose metabolism, but little is known about metabolic changes in precancerous skin lesions such as actinic keratosis (AK). Here, we studied the central carbon metabolism and immune cell infiltrate of actinic keratosis lesions before, under, and 4 weeks after treatment with topical diclofenac (Solaraze®).

**Methods:** This study was designed as a prospective, randomized, controlled, monocentric investigation (ClinicalTrials.gov Identifier: NCT01935531). Myeloid and T cell infiltration was analyzed in skin biopsies from 28 patients by immunohistochemistry. Furthermore, immune cell activation was determined via quantitative real-time PCR (*IFN-*γ, IL-10, *CSF1, TGF-*β*, IL-6*). Glucose, amino acid and Krebs' cycle metabolism was studied by mass spectrometry prior, during and after treatment with topical diclofenac. Biopsies from sun-exposed, untreated, healthy skin served as controls.

**Results:** Increased lactate and decreased glucose levels suggested accelerated glycolysis in pre-treatment AK. Further, levels of Krebs' cycle intermediates other than citrate and amino acids were elevated. Analysis of the immune infiltrate revealed less epidermal CD1a+ cells but increased frequencies of dermal CD8+ T cells in AK. Treatment with diclofenac reduced lactate and amino acid levels in AK, especially in responding lesions, and induced an infiltration of dermal CD8+ T cells accompanied by high *IFN-*γ mRNA expression, suggesting improved T cell function.

**Discussion:** Our study clearly demonstrated that not only cancers but also pre-malignant skin lesions, like AK, exhibit profound changes in metabolism, correlating with an altered immune infiltrate. Diclofenac normalizes metabolism, immune cell infiltration and function in AK lesions, suggesting a novel mechanism of action.

## Introduction

Actinic keratosis (AK) is a skin lesion characterized by disordered keratinocyte proliferation due to excessive ultraviolet (UV) exposure. It is regarded as an *in situ* squamous cell carcinoma as it can progress to invasive squamous cell carcinoma (1).

Prevalence of AK is high, especially in older individuals. Epidemiologic studies in Germany demonstrated a prevalence of 11.5 % among 60–70-year old patients (2). In the Netherlands, the prevalence is even higher with 49.0 and 28.1% of men and women with a mean age of 72 years suffering from one or more AK lesions (3). A similar high prevalence of AK is found in Australia, where 52% of men older than 70 years are affected (3, 4).

Topical diclofenac is a standard treatment modality for AK. However, the mechanism of action is not fully understood yet (5, 6). Inhibition of cyclooxygenase (COX-1/-2) and induction of apoptosis are discussed as possible underlying mechanisms (7–11).

We demonstrated inhibitory effects of diclofenac on tumor cell proliferation that were not related to COX inhibition (12). Diclofenac inhibited the oncogene MYC and significantly decreased glucose transporter-1 (GLUT-1), lactate dehydrogenase A (LDHA), and monocarboxylate transporter-1 (MCT-1) gene expression in line with a decrease in lactate secretion and glucose uptake. Of importance, in a murine glioma model, diclofenac treatment diminished intratumoral lactate levels, delayed glioma growth and rescued IL-12 production of tumor-infiltrating dendritic cells (DCs) (13).

Increased glycolysis under normoxia, known as the “Warburg effect” (14), enables tumor cells to synthesize building blocks such as non-essential amino acids and nucleotides for protein and nucleic acid biosynthesis. Moreover, this phenotype induces a highly immunosuppressive metabolic milieu with low glucose and high lactate levels (15). Several studies demonstrated strong effects of lactic acid on immune cell populations *in vitro* and *in vivo* (16). *In vitro*, administration of lactic acid inhibited proliferation and activation of human cytotoxic CD8 T cells, while LDHA-mediated production of lactate in tumor cells constrained IFN-γ production in tumor-infiltrating T cells, resulting in a loss of immune surveillance in a mouse melanoma model (17, 18). Furthermore, activation and antigen expression of human dendritic cells was suppressed by lactic acid *in vitro* (19). Accordingly, high intratumoral concentrations of lactate correlate with decreased patient survival in cancers such as head-and-neck cancers and melanoma (18, 20).

This shift to glucose metabolism results in diminished citrate synthesis and, thus, availability of acetyl-CoA, which in turn contributes to cancer aggressiveness via changes in the acetylation state of proteins (21). Likewise, changes in cancer amino acid metabolism can affect tumor infiltrating immune cells (15, 16). However, no data are currently available on glucose and amino acid metabolism in AK.

In this study, we examined the metabolism and the immune infiltrate of AK in response to topical diclofenac treatment.

## Materials and Methods

### Study Design

This study was designed as a prospective, randomized, controlled, monocentric investigation, with patients acting as their own control. The study was approved by the Institutional Review Board and the Ethics Committee of the University of Regensburg as well as by the German Federal Institute for Drugs and Medical Devices. Written informed consent had been obtained from each patient before enrolment. The number of patients required for this trial had been calculated during a prestudy statistical consultation (F. Zeman, M. Koller, University Hospital Regensburg). The trial was registered prior to the start of the study at clinicaltrials.gov (ClinicalTrials.gov Identifier: NCT01935531).

### Inclusion and Exclusion Criteria

The main inclusion criteria were oral and written informed patient consent; Caucasian male or female patients aged ≥18 years; a negative pregnancy test in women of childbearing age; clinical diagnosis of AK and a minimum of 3 AK lesions (Table 1).

**Table 1:** Flow diagram of the study.

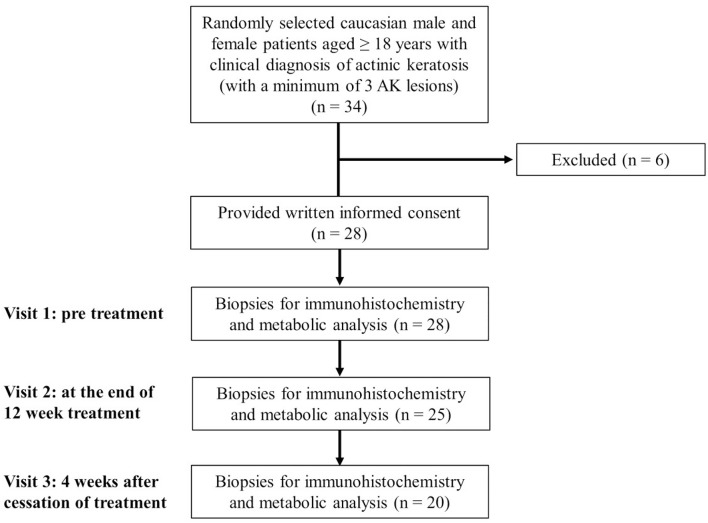

The main exclusion criteria were concomitant UV phototherapy, pregnancy or lactation, women of child-bearing age, who did not use highly efficient contraceptive methods, skin diseases that may interfere with the response evaluation of the study treatment, Fitzpatrick skin type IV-VI, any topical AK treatment of the dorsal hands during the 4 weeks preceding study treatment, topical or systemic treatment with retinoids, systemic treatment with cytostatic drugs during the 3 months preceding study therapy, any known intolerance to diclofenac or to any other ingredient of Solaraze® 3% gel (Almirall Hermal GmbH, Reinbek, Germany), systemic treatment with diclofenac, conditions that may interfere with a patient's ability to understand the study and give written informed consent, simultaneous participation in another clinical study or participation in another clinical study in the 30 days directly preceding inclusion, and suspected lack of compliance.

### Study Treatment

At visit 1, three AKs were randomly determined and documented as target lesions. If there were only 3 available AKs, all lesions were numbered consecutively (1–3). If more than 3 AKs were available, preferably similar AKs were chosen as target lesions and additional AKs were also documented and generally called study lesions. The first target lesion was biopsied (4–5 mm punch) prior to treatment at visit 1 after local anesthesia with Mepivacain (Mecain® 1%, Actavis, Munich, Germany). Another skin biopsy (target lesion number 2) was biopsied at the end of the 12-week treatment period. Target lesion number 3 was biopsied 4 weeks after cessation of treatment (Table 1). A control biopsy in sun damaged, untreated, healthy skin (e.g., capillitium, arms, legs) was additionally obtained in 10 suited patients upon patient's informed consent at visits 1 and 3. Biopsied tissues were immediately wraped in aluminum foil, snap-frozen in liquid nitrogen and stored at −80°C. This procedure did not exceed 1 min.

Three percent diclofenac in 2.5% hyaluronic acid gel (Solaraze® 3% gel) was applied 1 cm beyond the single AK twice daily on the study lesions. The localization and the size of the AK study lesions were documented in a chart in the CRF. Photographs from the treatment area were taken. Study participants were advised to avoid direct sun exposure in the treatment area.

### Clinical Evaluation

At visits 2 and 3, treatment efficacy was evaluated. Complete clearance was documented if a lesion was no longer visible and also imperceptible to the touch. Partial remission was documented if a lesion was clinically improved compared to photographs taken before treatment start. No remission was documented in case of not improved lesions. In case of small lesions that had been largely removed by the biopsy, no evaluation of treatment efficacy was possible.

### Histological and Immuno-Histochemical Analysis

The sections were stained following standard protocols for hematoxylin-eosin (HE) and evaluated by a dermatopathologist. AKs were graded as follows: In grade AK I atypical keratinocytes were restricted to the lower third of the epidermis. In grade AK II, atypical keratinocytes extended to the lower two-thirds of the epidermis. In grade AK III, full thickness atypia of the epidermis was found (22).

For immuno-histochemical staining with CD1a, CD4, CD8, CD68, and GLUT-1 paraffin-embedded skin samples were stained and evaluated by a blinded laboratory technician as described previously (23). The following antibodies were used: rabbit anti-human monoclonal anti-CD1a (clone EP3622), rabbit anti-human monoclonal anti-CD4 (clone SP35), mouse anti-human CD8 (clone C8/144B), rabbit anti-human monoclonal CD68 (clone SP251) and rabbit anti-human monoclonal GLUT-1 (clone SP168). All antibodies were purchased at Biozol, Eching, Germany. The selected pixels of the evaluated images were expressed as the percentage of the total area.

### Quantitative Real-Time PCR (qRT-PCR)

Snap-frozen biopsies were homogenized using TissueLyser and QIAshredder columns (Qiagen, Hilden, Germany). Subsequent isolation of total cellular RNA was performed using the RNeasy Mini Kit (Qiagen). RNA quantification and control of integrity was measured on RNA Nano LabChips using the 2100 Bioanalyzer (Agilent Technologies, Santa Clara, CA, USA). Complementary DNA was synthesized with a M-MLV Reverse Transcriptase kit (Promega, Madison, WI, USA) and was amplified by qPCR with the QuantiFast SYBR Green PCR Kit (Qiagen) using the Mastercyler Ep Realplex (Eppendorf, Hamburg, Germany). The following primers were used for human: *GLUT-1*, 5′-AACTCTTCAGCCAGGGTCCAC-3′ and 5′-CACAGTGAAGATGATGAAGACGTAGGG-3′; *LDHA*, 5′-GGTTGGTGCTGTTGGCATGG-3' and 5′-TGCCCCAGCCGTGATAATGA-3′; *LDHB*, 5′-GATGGTGGTTGAAAGTGCCTATGAAGTC−3′ and 5′-AGCCACACTTAATCCAATAGCCCA-3′; *CSF1*, 5′-CGAGCAGGAGTATCACCGAGGA-3′ and 5′-ATGTAATTTGGCACGAGGTCTCCATCTG-3′; *COX1*, 5′-CTACGAGCAGTTCTTGTTCAACACC-3′ and 5′-ATGACATCCACAGCCACATGCAG-3′; *COX2*, 5′-CCAGAGCAGGCAGATGAAATACCAG-3′ and 5′-TCGATGTCACCATAGAGTGCTTCC-3′; *TGF*β, 5′-CAGCAACAATTCCTGGCGATA-3′ and 5′-ATTTCCCCTCCACGGCTCAA-3′; *IL10*, 5′-GCAACCTGCCTAACATGCTTCGAG-3′ and 5′-CTGGGTCTTGGTTCTCAGCTTGGG-3′; *IL6*, 5′-TGCTTCCAATCTGGATTCAATGAGG-3′ and 5′-GCTCTGGCTTGTTCCTCACTACTC-3′ and *IFN*γ, 5′-CTAATTATTCGGTAACTGACTTGA-3′ and 5′-ACAGTTCAGCCATCACTTGGA-3′. PCR results were normalized to the control gene *18S*, 5′-ACCGATTGGATGGTTTAGTGAG-3′ and 5′-CCTACGGAAACCTTGTTACGAC-3′.

### Determination of Metabolites

Tissue specimens were weighted and homogenized in 600 μL of aqueous methanol (80:20, v/v) three times at 6,500 rpm for 30 s each with a 30-s pause in-between using the Precellys tissue homogenizer and Precellys vials filled with 1.4-mm ceramic beads (CK14 Soft Tissue Homogenizing Kit, Bertin Instruments, Montigny-le-Bretonneux, France). During homogenization, 40 μL of an internal standard mix containing stable isotope labeled analogs of the analytes were added. Subsequently, samples were centrifuged at 9560 × g for 5 min at 4°C and the supernatant was transferred to a 1.5-mL glass vial. The pellet was washed twice with 200 μL of 80% methanol, centrifuged at 13800 × g for the last wash, and all supernatants were combined. The combined extracts were then evaporated to complete dryness and reconstituted in 100 μL of water containing 0.1% formic acid. The extracts were centrifuged to obtain a clear sample and a 50-μL aliquot was taken for subsequent analysis of tryptophan metabolites and amino acids. The remaining extract was evaporated to complete dryness for GC-MS analysis of glucose, organic acids, and diclofenac.

Tryptophan metabolites were measured in the extracts by liquid chromatography-tandem mass spectrometry (HPLC-MS/MS) as previously described (24). Amino acids were analyzed by HPLC-MS/MS after derivatization with propyl chloroformate using a 10-μL sample aliquot as described previously (25).

Analysis of glucose, diclofenac, glycolysis and TCA cycle intermediates was performed by GC-MS. The dried sample residue was subjected to methoximation and silylation and subsequent GC-MS analysis using the derivatization protocol and instrumental setup previously described (26). An injection volume of 1 μL and splitless injection was employed. Starting at 50°C, the GC oven temperature was ramped first at 5°C/min to 120°C and, then, at 8°C/min to 300°C, where it was held for 5 min. Quantification was performed using a calibration curves for each analyte with the corresponding stable isotope labeled analog serving as internal standard.

### Statistical Analysis

Statistics were performed using the GraphPad 7 software. Significances were determined by Mann-Whitney and Kruskal-Wallis test.

## Results

### Increased Metabolic Activity in Actinic Keratosis Lesions

A total of 28 patients with a mean age of 72.9 ± 8.4 years and histologically confirmed AK were included in the study. Thirteen patients (46.4%) were diagnosed with grade I AK, while fourteen (50%) and one patient (3.6%) had grade II and III AK, respectively.

**Figure 1:**
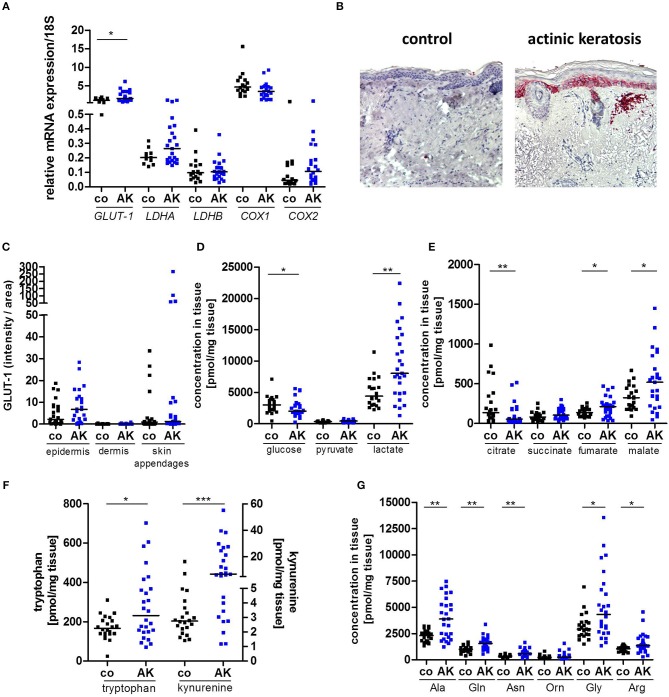
Increased lactate and amino acid levels in actinic keratosis lesions. **(A)** qRT-PCR analysis of *GLUT-1, LDHA, LDHB, COX1*, and *COX2* gene expression in actinic keratosis lesions (AK) and control biopsies (co) of sun-exposed, untreated, healthy skin in 28 patients. **(B)** Representative staining of GLUT-1 in actinic keratosis lesion and control biopsy. **(C)** Immunohistochemical staining of GLUT-1 in epidermis, dermis and skin appendages in actinic keratosis lesions and control biopsies. The selected pixels of the evaluated images were expressed as the percentage of the total area. **(D–G)** Determination of intratumor metabolite levels by mass spectrometry. The dark line indicates the median. *p < 0.05, **p < 0.01, ***p < 0.001 (Mann-Whitney test). Ala, alanine; Gln, glutamine; Asn, asparagine; Orn, ornithine; Gly, glycine; Arg, arginine.

Gene expression of *glucose transporter-1* (*GLUT-1*) was significantly enhanced in AK lesions compared to normal, sun-exposed skin controls (Figure 1A). Protein expression in epidermis was also tentatively increased (Figures 1B,C), but did not reach a statistically significant level (*p* = 0.1352). No significant differences in gene expression were detected for *lactate dehydrogenase A* (*LDHA*) and *LDHB*; likewise, the ratio between *LDHA* and *LDHB* was not altered (Figure 1A, Supplemental Figure 1A). As diclofenac blocks cyclooxygenase (COX) activity and COX-1 and−2 are overexpressed in many tumor entities (27), we also analyzed the expression of COX-1 and−2 in AK biopsies. Neither *COX-1* nor *COX-2* mRNA expression was altered in AK (Figure 1A), but the ratio between *COX-1* and *COX-2* was significantly decreased in AK lesions suggesting a predominance of the “tumor-specific” *COX-2* in AK (Supplemental Figure 1B).

Although GLUT-1 protein was not upregulated, significantly decreased glucose levels and a 2-fold increase in lactate levels were detected in pre-malignant AK lesions (Figure 1D), consistent with accelerated glycolytic activity. Interestingly, TCA metabolites were dysregulated in AK. The tissue concentrations of citrate were significantly reduced in AK lesions compared to controls, while those of fumarate and malate were significantly increased (Figure 1E).

We also determined amino acid levels, as tryptophan, arginine or glutamine metabolism can undergo profound changes in tumors (16). Levels of both kynurenine and tryptophan were increased in AK, and so was the kynurenine-to-tryptophan ratio (Figure 1F, Supplemental Figure 1C), which indicates increased indoleamine 2,3-dioxygenase (IDO) activity. Additionally, AK exhibited higher concentrations of almost all amino acids with the exception of ornithine and methionine (Figure 1G, Supplemental Figure 1D).

In summary, AK lesions showed the characteristic metabolic signs of tissues sustaining increased proliferation, namely increased glycolysis and uptake of amino acids. The increased levels of fumarate and malate in the presence of decreased levels of citrate suggest anaplerosis to sustain amino acid biosynthesis and redox homeostasis.

### Reprogramming of AK Metabolism After Treatment With Diclofenac

AK lesions were treated for 3 months with topical diclofenac (Solaraze®) and then observed for another 4 weeks to record response to treatment. They were devided based on the response to diclofenac treatment to responders (normal skin 4 weeks after treatment) and non-responders (persistent AK 4 weeks after treatment). In both groups similar diclofenac levels could be detected in AK biopsies (data not shown).

**Figure 2:**
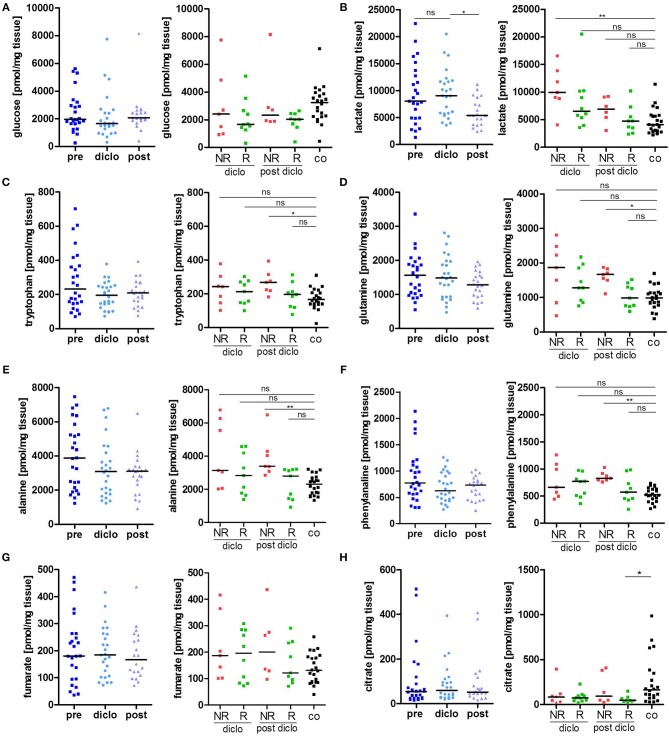
Metabolic response to diclofenac in AK lesions. **(A–H)** Temporal changes in intratumor metabolite levels of **(A)** glucose, **(B)** lactate, **(C)** tryptophan, **(D)** glutamine, **(E)** alanine, **(F)** phenylalanine, **(G)** fumarate, and **(H)** citrate, in actinic keratosis lesions and control skin biopsies. Left graphs show all patients pre, on (diclo), and post treatment with diclofenac. Right graphs show non-responders (NR, *n* = 8) and responders (R, *n* = 12) to diclofenac on (diclo) and post treatment compared to controls (co). The dark lines indicate the median. *p < 0.05, **p < 0.01 (Kruskal-Wallis test).

Treatment of AK lesions with diclofenac for 12 weeks did not affect glucose tissue levels (Figure 2A). There was a slight, but insignificant increase in glucose tissue levels 4 weeks after cessation of treatment. There were also no significant differences in glucose tissue levels between responders and non-responders. While overall levels of lactate in AK lesions did not change under treatment with diclofenac compared to levels before initiation of treatment, there was a drop 4 weeks after completion of treatment (Figure 2B). However, subgroup analysis showed, that responders had already experienced a strong reduction of lactate levels 12 weeks into the treatment with diclofenac. After treatment, also non-responders reached lactate levels comparable to the control indicating a delayed response (Figure 2B).

Tryptophan levels were not significantly decreased during and after treatment with diclofenac and reached control levels in responding lesions while non-responding lesions exhibited still elevated levels after treatment (Figure 2C). The kynurenine-to-tryptophan ratio reached control levels in both non-responders and responders (Supplemental Figure 1E). Accordingly, glutamine, alanine and phenylalanine levels and all other analyzed amino acids were decreased in responding lesions only and reached normal skin levels (Figures 2D–F, Table 2). No changes were observed in the glutamine/glutamate ratio (Supplemental Figure 1F). Notably, no significant changes in levels of Krebs' cycle intermediates fumarate, malate and succinate could be observed during and after treatment with diclofenac (Figure 2G, Table 2). Interestingly, citrate levels remained low in AK lesions compared to controls both during and 4 weeks after cessation treatment (Figure 2H).

**Table 2:** Decreased glucose, amino acid, and TCA metabolites in AK lesions responding to diclofenac.

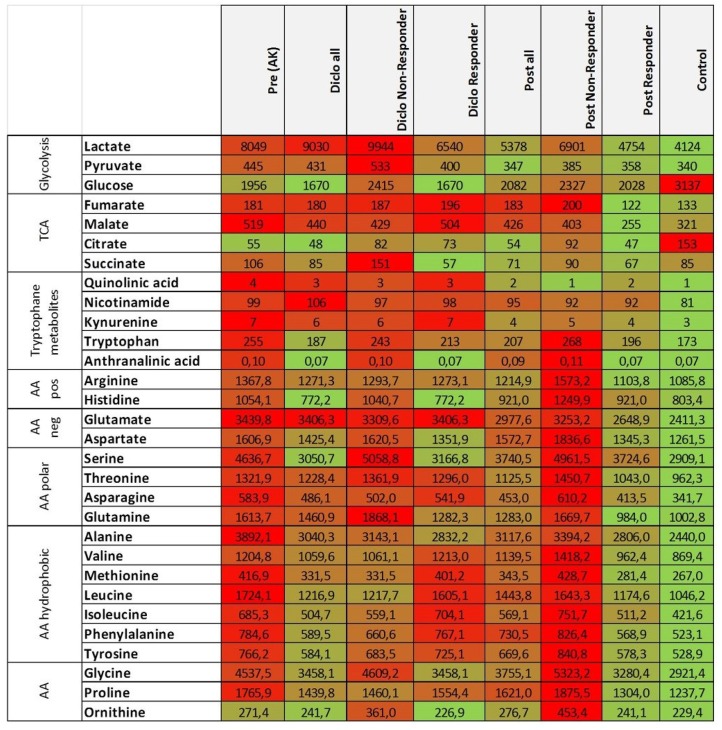

*Differences between levels of metabolic pathways between pre [pre (AK)], on (Diclo) and post treatment with diclofenac in all patients or, separately, in non-responders and responders to solaraze®. Data are medians of metabolite concentrations in pmol/mg tissue. Red color represents high concentrations, green color represents low concentrations of metabolites*.

In summary, these data suggest that diclofenac treatment impacts at least in part the metabolic state of AK lesions.

### Decrement of Epidermal Langerhans Cells in Actinic Keratosis Lesions

**Figure 3:**
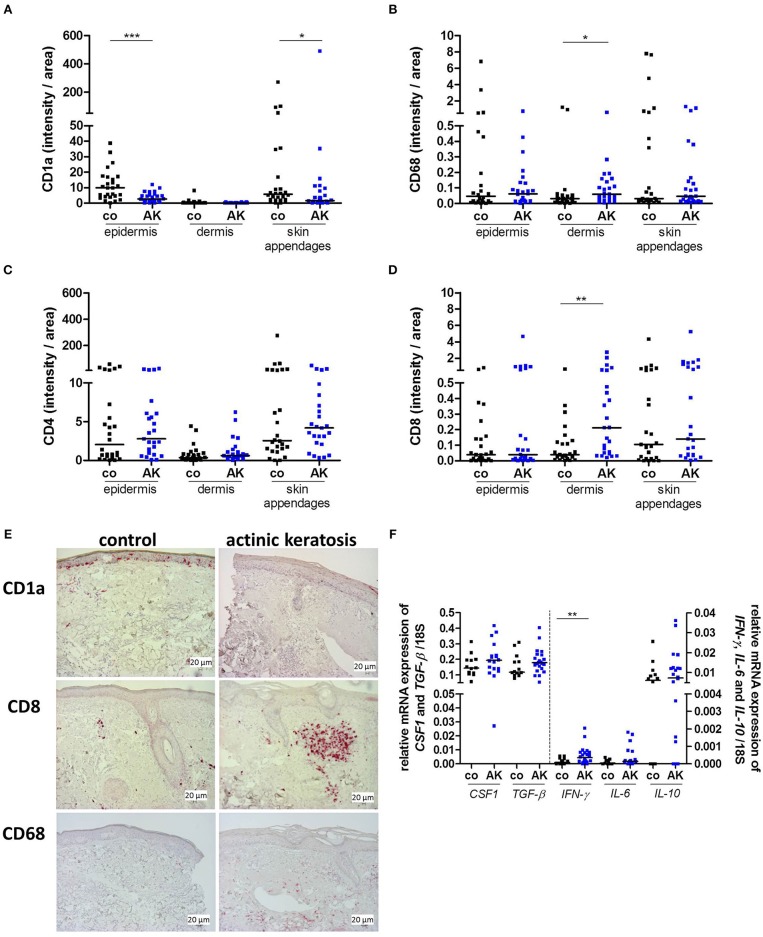
Actinic keratosis induces changes in the skin immune infiltrate. **(A–D)** Immunohistochemical staining of **(A)** CD1a, **(B)** CD68, **(C)** CD4, and **(D)** CD8 in epidermis, dermis and skin appendages in actinic keratosis lesions (AK) and control biopsies (co) of sun-exposed, untreated, healthy skin in 28 patients. The selected pixels of the evaluated images were expressed as the percentage of the total area. **(E)** Representative stainings of CD1a (upper images), CD8 (middle images), and CD68 (lower images) in actinic keratosis lesions and control biopsies. **(F)** qRT-PCR analysis of *CSF1, TGF-*β, *IFN-*γ, *IL-6, and IL-10* gene expression in actinic keratosis lesions and control skin biopsies. The dark lines indicate the median. *p < 0.05, **p < 0.01, ***p < 0.001 (Mann-Whitney test).

We examined whether the immune cell composition in AK is altered as compared to control biopsies. To this end, markers defining typical immune cell subsets in skin such as Langerhans cells (LC, CD1a), macrophages (CD68), and T cells (CD4, CD8) were stained immunohistochemically. While less CD1a+ LCs were detected in the epidermis and skin appendages of patients with AK, no differences were found in the dermis (Figures 3A,E). CD68+ macrophages were located in epidermis, dermis and skin appendages and a higher frequency of macrophages could be detected in the dermis of AK (Figures 3B,E).

LCs play a key role as immune sentinels at the skin barrier surface as they act as antigen-presenting cells and regulate differentiation and effector functions of T cells. Therefore, we next examined skin infiltrating T cells. CD4+ T cells were more prominent than CD8+ T cells in all dermal layers. No difference in CD4+ T cell infiltration between epidermis, dermis, skin appendages and neither between control biopsy and AK was observed (Figure 3C). Interestingly, CD8+ T cells infiltrated into the dermis of AK, whereas only a few CD8+ T cells were present in healthy skin (Figures 3D,E). Beside surface markers, immune cells can be characterized by functional parameters such as cytokine expression. To further describe the immune infiltrate of AK, we analyzed cytokines that are secreted by T cells and/or myeloid cells and are involved in the regulation of inflammation. The expression of *colony stimulating factor 1* (*CSF1*), *transforming growth factor* β (*TGF-*β) as well as *interleukin-10* (*IL-10*), and *-6* (*IL-6)* was not changed in AK (Figure 3F) in comparison to healthy skin but *interferon-*γ (*IFN-*γ*)*the most prominent anti-tumoral T cell (and NK cell) cytokine (28), was significantly elevated in AK (Figure 3F).

### Diclofenac Counteracts Immune Cell Alterations in AK Lesions

**Figure 4:**
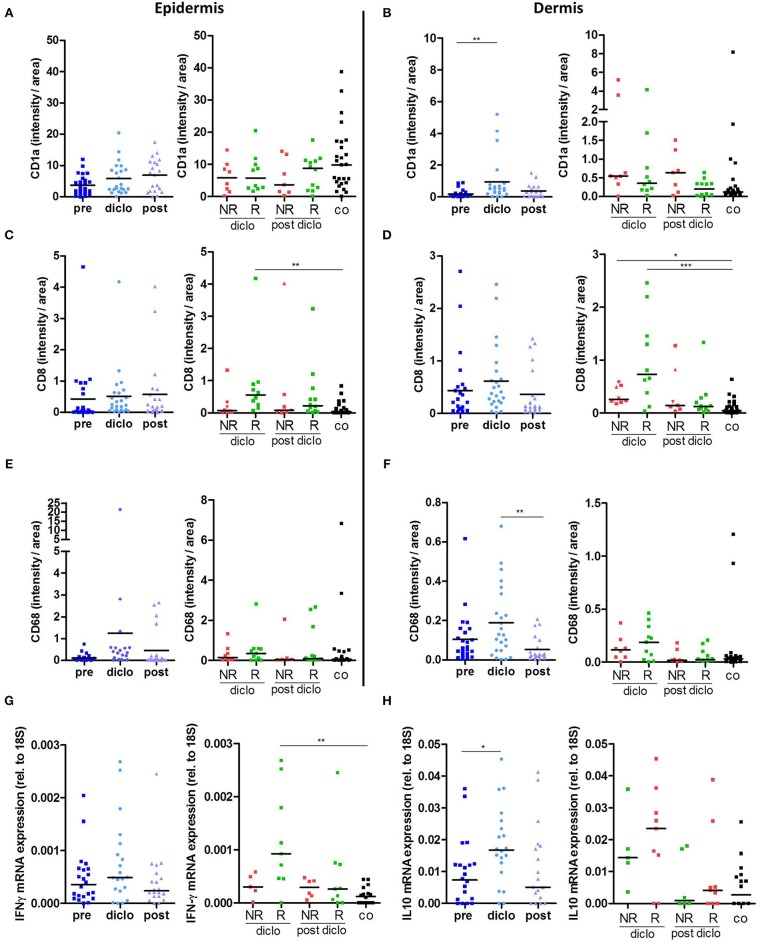
Diclofenac induced an increased immune response. **(A–E)** Immunohistochemical staining of **(A,B)** CD1a, **(C,D)** CD8, and **(E,F)** CD68 in epidermis and dermis in actinic keratosis lesions (AK) and control skin biopsies (co). The selected pixels of the evaluated images were expressed as the percentage of the total area. **(G,H)** qRT-PCR analysis of **(G)** *IFN-*γ and **(H)** *IL-10* gene expression in actinic keratosis lesions and control skin biopsies. Left graphs show all patients pre, on (diclo) and post treatment with diclofenac. Right graphs show non-responders (NR, *n* = 8) and responders (R, *n* = 12) to diclofenac on (diclo) and post treatment compared to controls (co). The dark lines indicate the median. *p < 0.05, **p < 0.01, ***p < 0.001 (Kruskal-Wallis test).

Our previous studies revealed that diclofenac has a strong impact on metabolism and immune cell infiltration in tumors (13). Therefore, we examined whether diclofenac also influenced the immune cell composition in AK lesions. As already shown in Figure 3A, CD1a expressing LCs were markedly reduced in precancerous AK lesions compared to healthy, sun-exposed, untreated skin. Application of diclofenac increased the number of epidermal CD1a+ cells in responders by trend (Figure 4A, Supplemental Figure 2) and elevated dermal infiltrating CD1a+ cells (Figure 4B, Supplemental Figure 2).

Of interest, the number of CD8+ T cells in the epidermis as well as in the dermis increased in responders during treatment indicating that diclofenac promoted CD8+ T cell infiltration in these skin layers (Figures 4C,D, Supplemental Figure 2). In the dermis, also an infiltration of CD8+ T cells in non-responding lesions could be observed (Figure 4D, Supplemental Figure 2).

In contrast to CD8+ T cells, we did not detect differences in the frequency of CD4+ T cells, neither in the epidermis nor in the dermis of patients with AK before, during and after the treatment (data not shown). The level of dermal CD68+ macrophages decreased after diclofenac treatment (Figures 4E,F, Supplemental Figure 2).

Next, we examined local cytokine and growth factor levels during and after treatment with diclofenac. Interestingly, *IFN-*γ mRNA expression was significantly elevated during treatment with diclofenac in responders (Figure 4G), which correlated with higher CD8+ T cell infiltration (Figures 4C,D). Additionally, *IL-10* mRNA expression levels were increased in AK lesions indicating the presence of an anti-inflammatory reaction (Figure 4H). Expression of IL-6 was enhanced by trend in non-responding lesions during treatment with diclofenac, while expression levels of *CSF1* was not altered (data not shown). *TGF-*β levels were reduced in all biopsies after treatment with diclofenac but no differences between responding and non-responding lesions could be detected (data not shown).

Therefore, treatment with diclofenac led to a strong infiltration of dermal CD8+ T cells along with an increase in type II IFN and IL-10 expression.

## Discussion

Diclofenac, a nonsteroidal anti-inflammatory drug (NSAID), is used for the field treatment of AK lesions. However, its mode of action is not fully understood. Inhibition of cyclooxygenase (COX-1/-2) and, consequently, angiogenesis and cellular proliferation is a proposed mechanism of action (7, 8). Also, COX-independent mechanisms, such as the induction of apoptosis by NSAIDs, are discussed (9–11). Interestingly, COX-2 inhibition is discussed to elicit anti-proliferative response in human cancer cell lines via induction of endoplasmatic reticulum stress (29). So far, no clinical data exist on the impact of diclofenac treatment on glucose and amino acid metabolism.

Accelerated glucose metabolism is a well-known feature of melanomas as the BRAF oncogene causes the upregulation of genes involved in glycolysis (30). Increased GLUT-1 expression could also be detected in BRAF wild-type skin lesions, such as squamous cell carcinomas (SCC) and AK (31) indicating that increased glucose metabolism contributes to tumor development of cutaneous neoplasia. Analyses of AK biopsies revealed high levels of lactate accompanied by increased *GLUT-1* mRNA expression and decreased glucose levels. While diclofenac did not influence glucose levels in either responding or non-responding AK, the lactate concentration was significantly reduced in AK lesions after treatment. This is in line with our previous findings showing that diclofenac inhibits lactate secretion in glioma and leukemia cell lines (12, 13, 32). Of importance, responding lesions exhibited reduced lactate levels already during therapy, whereas non-responding lesions exhibited decreased lactate levels after therapy. In these patients, an elongation of the therapy with diclofenac might further reduce lactate levels in these highly metabolic AK. In line, Ulrich et al. and Nelson et al. reported a better response to diclofenac therapy after longer treatment (11, 33).

High intratumoral lactate concentrations correlate with decreased patient survival in head-and-neck tumors and melanoma patients (18, 20) and limit T cell function. Additionally, accelerated glucose metabolism can lead to glucose deprivation resulting in diminished T cell effector functions (34, 35). Based on the profound metabolic changes in AK lesions, we analyzed immune cell infiltration during treatment and observed a strong infiltration of epidermal and dermal CD8+ T cells in responding AK, accompanied with strong mRNA expression of IFN-γ. In contrast, another publication showed an NSAID mediated inhibition of IFN-γ secretion in NK cells and γδ-T cells (36). Cytotoxic CD8+ T cells play a pivotal role in the elimination of virus-infected and malignant cells (37, 38) and for most cancers, an infiltration with cytotoxic T cells is associated with a good prognosis (28). In our previous work, we clearly demonstrated in a murine B16 melanoma model, that inhibition of lactate production by LDHA downregulation improves immunosurveillance and restrains tumor in an IFN-γ dependent manner (18). Strong infiltration with CD8+ T cells was also described after topical treatment with ingenol mebutate, a standard drug for AK (39, 40), but in this study the number of CD4+ T cells and myeloid cells was also increased, suggesting an unspecific inflammatory reaction.

IL-10 was also enhanced in AK lesions during treatment with diclofenac. The principal function of IL-10 is the limitation of inflammatory responses, but it is also able to regulate the growth and differentiation of several cell types, namely immune cells, endothelial cells as well as keratinocytes (41). We hypothesize, that IL-10 is produced by infiltrating macrophages or immunosuppressive T cell populations and represents a possible mechanism to suppress the immune response in AK.

We detected significantly less CD1a expressing Langerhans cells (LC) in the epidermis of AK. After the treatment with diclofenac, responders show by trend a normal skin infiltrate with higher numbers of LCs. Interestingly, in a murine glioma model, diclofenac also resulted in higher frequencies of intra-tumoral CD11c+CD86+ DCs (13). Of importance, these DCs were also more activated and produced higher amounts of IL-12 which in turn could lead to the activation of T cells. This phenomenon might have occurred in our study and could explain the increased CD8+ T cell associated immune response.

Shevchuk et al. found decreased epidermal CD1a+ and CD207+ LCs in SCC and decreased numbers of CD207+ LCs in precancerous AK lesions (42). In contrast, a recent study described higher numbers of LCs in AK. This discrepancy might be due to the fact that (39) we used healthy sun-exposed skin as control whereas Emmert and co-workers selected non-sun-exposed skin. In line with other studies, our data show a clear dermal infiltration of CD68+ macrophages as well as CD8+ T cells in AK (39, 43).

Beside alterations in glucose metabolism with decreased glucose and increased lactate levels, we also detected higher concentrations of several amino acids in AK compared to untreated, sun-exposed skin. After treatment with diclofenac, lactate and almost all amino acids reached normal skin levels in responding biopsies. As glycolytic intermediates such as pyruvate are used to produce amino acids, accelerated glycolysis could partially explain increased amino acid levels in AK lesions. Precancerous AK lesions might require accelerated glucose and amino acid metabolism to synthesize building blocks and gain energy to allow rapid proliferation of keratinocytes (44). Recently, it has been discussed that targeting glutaminolysis is a possible approach to treat SCC. As AK is a precancerous lesion leading to SCC this approach may also be appropriate for treatment of AK (45).

Tryptophan levels were elevated in AK lesions but the kynurenine/tryptophan ratio reached control levels after treatment. Tryptophan is metabolized by indoleamine 2,3-dioxygenase (IDO), which catalyzes the conversion of tryptophan to kynurenine. IDO is expressed by different cell types and can lead to tryptophan depletion. Disturbed tryptophan metabolism and high expression of IDO have been described in different tumor entities, e.g., in esophageal SCC but also in patients with dermatitis or psoriasis (46, 47). Of importance, Hennequart et al. could show a COX-2 driven expression of IDO-1 in tumor cells and COX-2 inhibitors were able to diminish IDO-1 expression (48). Thus, diclofenac is able to affect tryptophan metabolism in AK.

In summary, we could clearly show that not only cancers but also pre-malignant skin lesions exhibit profound changes in metabolism, correlating with an altered immune infiltrate. Both, metabolism and immune infiltration are influenced by topical diclofenac treatment indicating that these alterations contribute to the mode of action beside the well-known inhibition of cyclooxygenase.

## Data Availability

The datasets generated for this study are available on request to the corresponding author.

## Ethics Statement

This study was designed as a prospective, randomized, controlled, monocentric investigation, with patients acting as their own control. The study was approved by the Institutional Review Board and the Ethics Committee of the University of Regensburg as well as by the German Federal Institute for Drugs and Medical Devices. Written informed consent had been obtained from each patient before enrolment. The number of patients required for this trial had been calculated during a prestudy statistical consultation (F. Zeman, M. Koller, University Hospital Regensburg). The trial was registered prior to the start of the study at clinicaltrials.gov (ClinicalTrials.gov Identifier: NCT01935531).

## Author Contributions

MK, ED, SK, and MB developed the study design and initiated the study. KS, ED, PU, GS, and KD collected data and performed initial analyses. KS, KP, KR, and PS did detailed analyses and created most data figures. MK, WH, and PO wrote the manuscript with editing assistance from all co-authors.

### Conflict of Interest Statement

The authors declare that the research was conducted in the absence of any commercial or financial relationships that could be construed as a potential conflict of interest.
