# Supplementary material for: Topical Diclofenac Reprograms Metabolism and Immune Cell Infiltration in Actinic Keratosis
Source: Front Oncol. 2019 Jul 3;9:605. doi: 10.3389/fonc.2019.00605 (PMC6619385; doi:10.3389/fonc.2019.00605)
Supplement: Supplementary file 1 [file Data_Sheet_1.PDF]

# SUPPLEMENTAL FIGURE 1

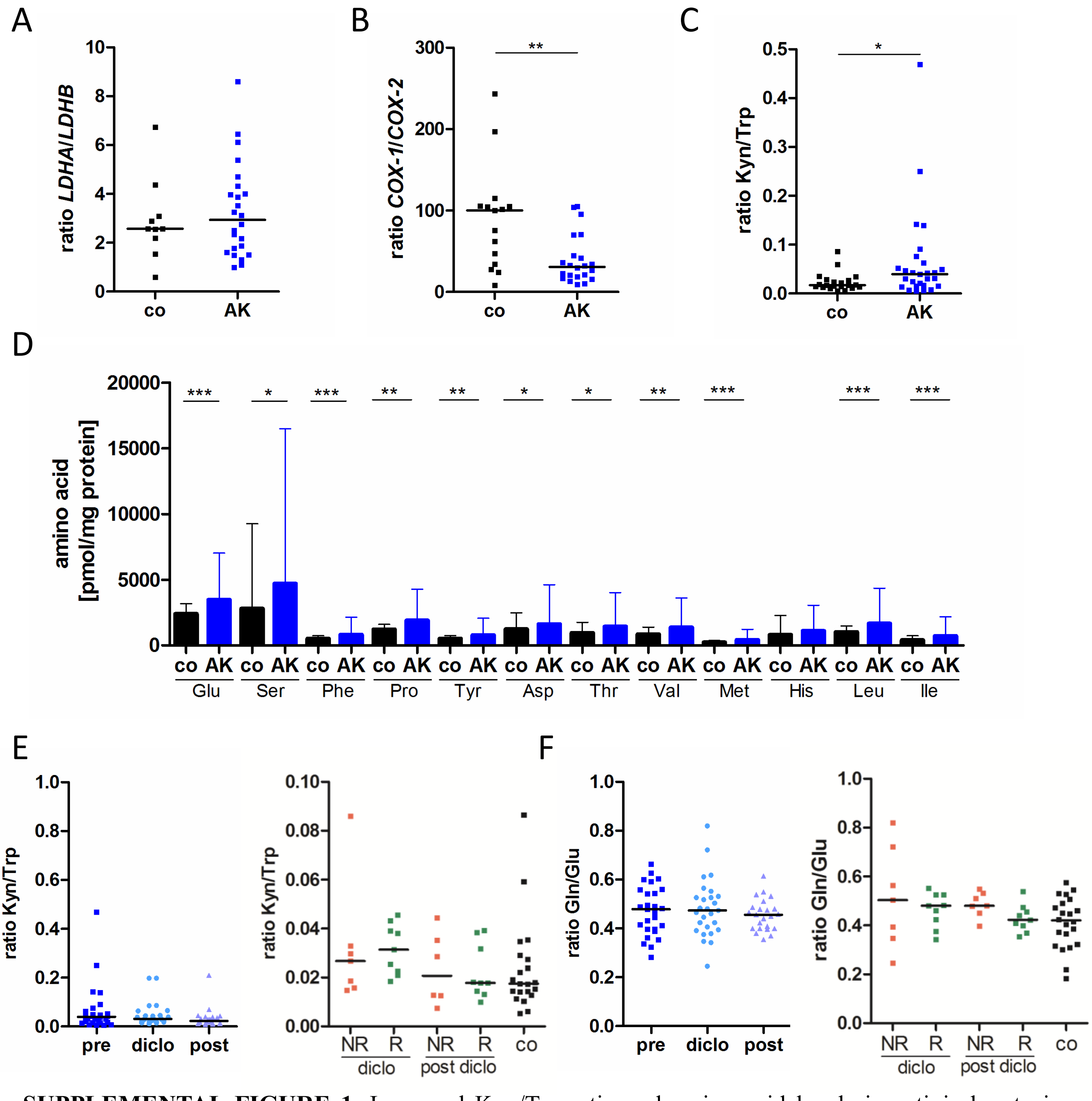


**SUPPLEMENTAL FIGURE 1.** Increased Kyn/Trp ratio and amino acid levels in actinic keratosis lesions. (**A, B**) qRT-PCR analysis of *LDHA, LDHB, COX1* and *COX2* gene expression in actinic keratosis lesions (AK) and control skin biopsies (co). Ratios between indicated enzymes are shown. **(C - F)** Quantification of intratumoral kynurenine/tryptophan and glutamine/glutamate ratios as well as levels of glutamate (Glu), serine (Ser), phenylalanine (Phe), proline (Pro), tyrosine (Tyr), aspartate (Asp), threonine (Thr), valine (Val), methionine (Met), histidine (His), leucine (Leu), and isoleucine (Ile) by mass spectrometry. **(C, E, F)** Ratios of kynurenine/tryptophan (Kyn/Trp) and glutamine/glutamate (Gln/Glu). **(E, F)** Left graphs show all patients pre, on (diclo) and post treatment with diclofenac. Right graphs show non-responders (NR, n = 8) and responders (R, n = 12) to diclofenac treatment compared to controls (co). The dark lines indicate the median. * $p < 0.05$, ** $p < 0.01$, ***$p < 0.001$ (Mann- Whitney and Kruskal -Wallis test).
