# Supplementary material for: Topical Diclofenac Reprograms Metabolism and Immune Cell Infiltration in Actinic Keratosis
Source: Front Oncol. 2019 Jul 3;9:605. doi: 10.3389/fonc.2019.00605 (PMC6619385; doi:10.3389/fonc.2019.00605)
Supplement: Supplementary file 2 [file Data_Sheet_2.PDF]

SUPPLEMENTAL FIGURE 2

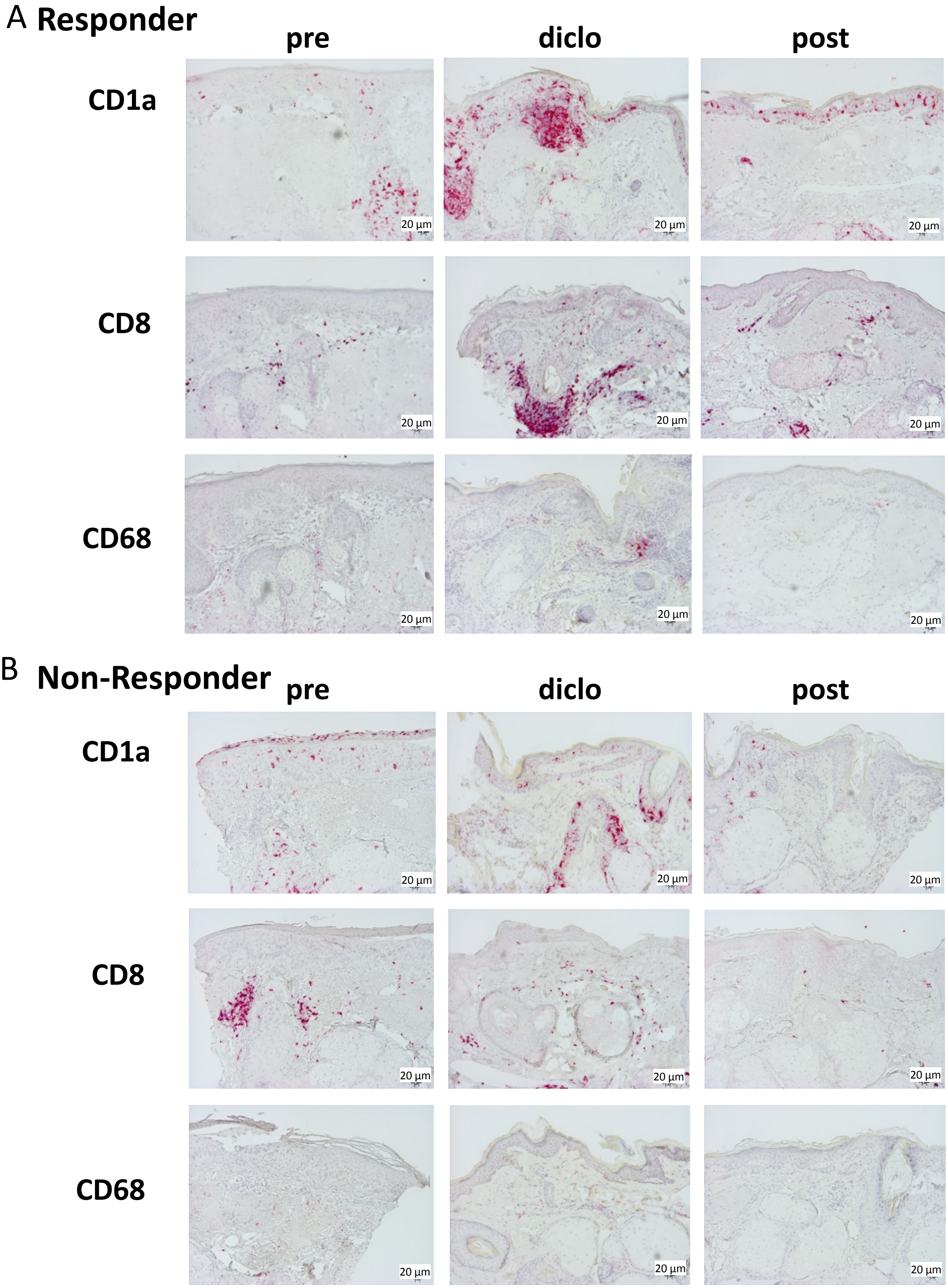

SUPPLEMENTAL FIGURE 2. Representative stainings of CD1a, CD8 and CD68 in actinic keratosis lesions pre, during (diclo) and post treatment with diclofenac of a (**A**) responder and a (**B**) non-responder.
